# Effectiveness and feasibility of a penicillin allergy delabeling program in the postacute inpatient rehabilitation setting

**DOI:** 10.1017/ice.2024.138

**Published:** 2024-11

**Authors:** Joseph Galipean, Jerry Jacob

**Affiliations:** 1 Hospital of the University of Pennsylvania, Philadelphia, USA; 2 Division of Infectious Diseases, Perelman School of Medicine, University of Pennsylvania, Philadelphia, USA

## Background

Penicillin (PCN) allergy is the most commonly reportedly drug allergy, and is reported by up to 20% of hospitalized patients.^1^ The overwhelming majority of these patients are shown to not have true IgE-mediated hypersensitivity reactions when assessed by penicillin skin testing (PST).^1^ Reported history of PCN allergy has been associated with increased morbidity including longer hospital stays, and increased rates of infection with drug-resistant organisms including *C. difficile*.^2,3^ PCN allergy delabeling programs can identify patients who may safely receive penicillin and related antibiotics, and thereby improve antibiotic utilization leading to better clinical outcomes, decreased antimicrobial resistance, and reduced costs.^2,3^ To our knowledge there have been no published reports of PCN allergy delabeling programs in the inpatient rehabilitation setting.

## Methods

A PCN delabeling program was implemented by the antibiotic stewardship team, including a clinical pharmacist and infectious diseases (ID) physician, at an inpatient rehabilitation facility associated with an academic medical center between 8/2020 and 10/2023. Inpatients with PCN allergies were identified weekly by manual review of the EMR. All patients with a PCN allergy were interviewed by the clinical pharmacist to assess for potential delabeling by history, or physician assessment for PST and/or direct oral challenge (DOC). Interviews were based on the 2019 American Medical Association’s toolkit.^4^ Patients were delabeled by history if they had a documented tolerance to a penicillin, or reported an intolerance instead of an allergy. Exclusion criteria for PST included antihistamine use, including TCAs, within the last 5 days, beta-blocker use within the last 2 days, immunosuppression, acute illness except in case of infection where beta lactam therapy is preferred 1st line agent, history of a high risk allergy, history of a positive PST or IgE-mediated reaction to PCN within the last 5 years, or inability to provide informed consent. Allergies were classified as high risk for severe non-IGE reactions, moderate-high risk for potential IgE reactions, and low risk for itching without rash and unknown/remote history without features of IgE reaction.^4^

PST used PRE-PEN as per package insert, penicillin G 10,000 units/mL, histamine 6 mg/mL positive control, and saline negative control. A prick test was performed initially. If negative after 15 minutes, an intradermal test was performed. Positive PST results were defined as wheal >3 mm over saline control for prick test, or wheal >2 mm over the original bleb for the intradermal test. A DOC would occur if the patient had a negative PST or the ID physician chose to forgo the PST. DOC consisted of either amoxicillin 250 mg PO × 1, or staged administration of amoxicillin 50 mg PO × 1 followed by amoxicillin 450 mg PO × 1.

## Results

Of the 145 patients that were evaluated, 9 (6.2%) reported a high risk allergy, 84 (57.9%) reported a moderate-high risk allergy, 45 (31.0%) reported had a low-risk allergy, and 7 (4.8%) reported an intolerance. Thirty (20.7%) patients were delabeled by history alone: 7 (4.8%) due to not having a true allergy, and 23 (15.9%) due to documented tolerance to penicillin. Seventy-one (49.0%) patients did not meet eligibility criteria for penicillin allergy skin testing: 63 (43.5%) were on a contraindicated medication, 5 (3.4%) had a recent IgE-mediated reaction, and 3 (2.0%) had a recent positive skin test. Of the 44 (30.3%) that were eligible: 36 (24.8%) refused intervention, and 8 (5.5%) were tested.

**Figure 1.**
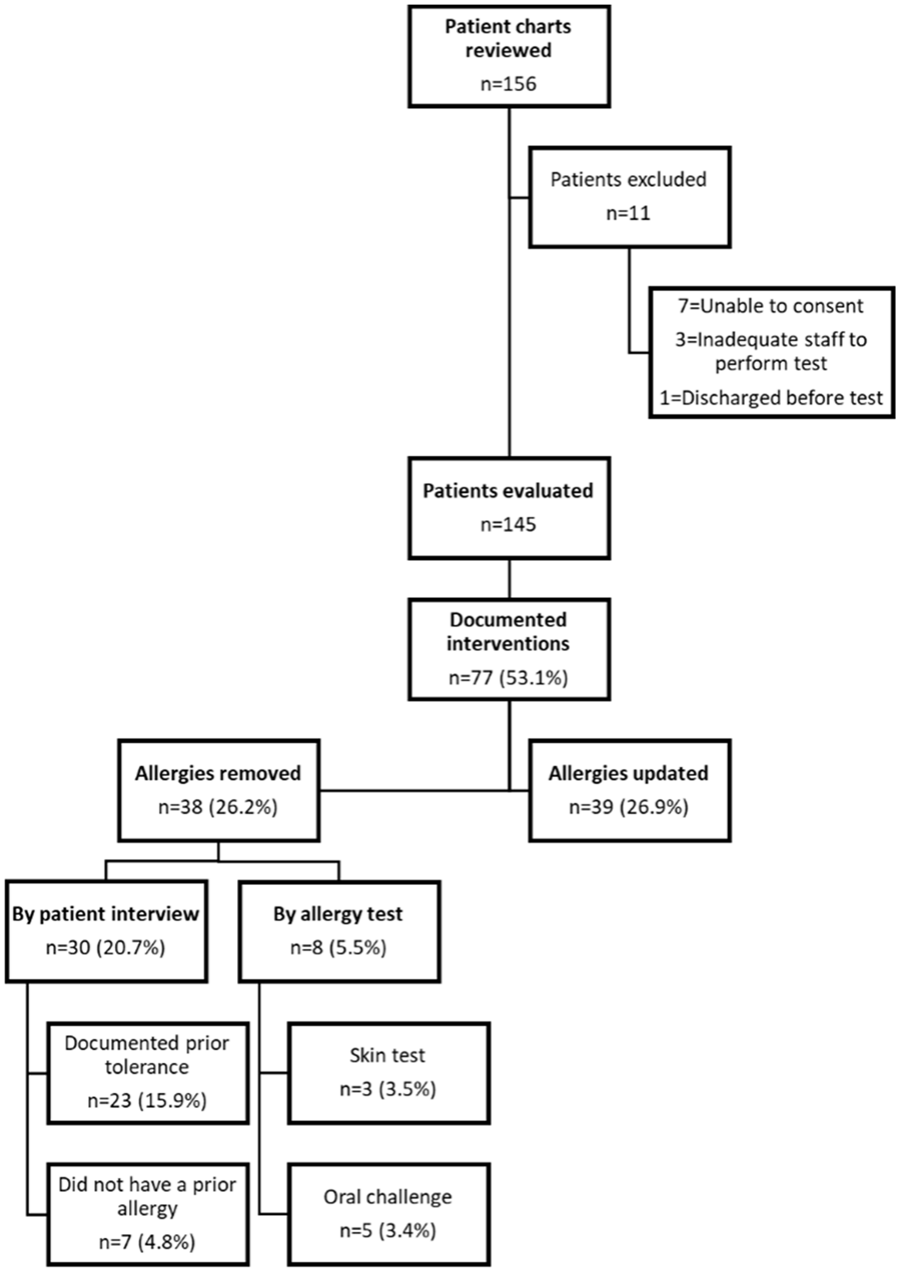
Results of interventions for patients labeled with a penicillin allergy in their chart.

## Discussion

We developed a program for proactive PCN allergy delabeling in the inpatient rehabilitation setting as it offered a clear opportunity to minimize preventable adverse outcomes with minimal resources (2 pharmacist hours weekly). Inpatient rehabilitation seems to be an opportune setting for PCN allergy evaluation as patients generally do not have acute medical issues, remain in a supervised care setting for an average of 2 weeks, and are likely at increased risk for antibiotic utilization given recent hospitalization. During our program, the Journal of Allergy and Clinical Immunology published updated drug allergy guidelines in December of 2022.^5^ These guidelines now also recommend proactive efforts at penicillin allergy delabeling.

Implementation of our PCN allergy delabeling program also uncovered limitations of this setting. As we did not have an allergist on staff or significant prior experience with penicillin skin testing, a conservative inclusion and exclusion criteria was used. In addition, the more recently published guidelines favor proceeding to DOC without PST in adults with low-risk penicillin allergy histories, and utilizing PST primarily for patients with a history of anaphylaxis or a recent reaction suspected to be IgE-mediated (eg, immediate onset urticaria).^5^ If this pilot was repeated with this updated approach, it is likely that more patients would have been captured and delabeled. Our inpatient protocol has accordingly been updated to allow for broader inclusion and increased use of direct oral challenge without preceding PST. The updated recommendation also decreases the cost associated with the program. Pre-Pen was the significate cost driver with a price ≈$194 compared to a DOC with a cost of <$1.^6^

In conclusion, this report provides support for PCN allergy delabeling programs in the postacute care setting. While many postacute units may not have an onsite ID physician or allergist to perform PST, our results suggest there may be opportunity for a substantial number of patients to be delabeled using standardized protocols for history-taking and direct oral challenge alone without specialist support. Increased adoption of PCN allergy delabeling programs in these settings may also have more widespread impact in acute and outpatient settings by decreasing adverse outcomes for patients mislabeled with a PCN allergy.
